# Twiddler’s Syndrome Causing Lead Fracture in Sacral Neuromodulation: A Case Report

**DOI:** 10.7759/cureus.56827

**Published:** 2024-03-24

**Authors:** Octavio Herrera, Maria V Rodriguez, Manuel R De Jesus, Alexis Garza, Henry Ruiz

**Affiliations:** 1 Urology, Doctor's Hospital at Renaissance Health, Edinburg, USA

**Keywords:** case report, lead migration, lead fracture, twiddler's syndrome, sacral neuromodulation

## Abstract

Twiddler’s syndrome is the voluntary or involuntary manipulation of an implanted device, most described in cardiac literature. Lead coiling may result in device malfunction due to lead migration or, less commonly, lead fracture. There are few but increasing reports of Twiddler’s syndrome resulting in lead migration in sacral neuromodulation, but lead fracture has not yet been described.

A 57-year-old Latina female presented with fecal incontinence and refractory overactive bladder. She underwent successful implantation of a sacral neuromodulation device with the resolution of symptoms. Following significant weight loss and two falls, she developed a recurrence of symptoms and was found to have lead migration on pelvic radiographs. At the time of surgical intervention, radiographs demonstrated worsened Twiddler’s syndrome with complete lead fracture despite no further trauma. She subsequently underwent partial lead removal and replacement with additional measures to prevent Twiddler’s syndrome and its sequelae.

Twiddler’s syndrome resulting in lead fracture can occur in sacral neuromodulation. Preventive techniques may be applied for patients with known risk factors for Twiddler’s syndrome, especially generator anchoring and lead replacement.

## Introduction

Twiddler’s syndrome (TS) is the manipulation of an implantable device that results in twisting of the attached lead, often causing device malfunction due to lead displacement or potentially lead fracture. This phenomenon is well-described in cardiac devices, such as pacemakers and cardioverter-defibrillators [1]. More recently, TS has been described in neuromodulator devices including deep brain and vagal nerve stimulators [2,3]. There are few reports of TS in the sacral neuromodulation literature, which describe lead migration and subsequent device failure resulting in symptom recurrence [4-7]. We present the first reported case of TS resulting in a lead fracture in sacral neuromodulation.

## Case presentation

A 57-year-old Latina female presented to the urology clinic due to fecal incontinence and refractory overactive bladder. Relevant past medical history included uncontrolled diabetes mellitus (initial glycated hemoglobin (HbA1C) 9.6%), coronary artery disease requiring percutaneous coronary intervention, and obesity with a body mass index of 38. She underwent a staged placement of a sacral neuromodulator (InterStim, Medtronic, Minneapolis, MN) with significant improvement of symptoms. She underwent an intentional 15-pound weight loss over the next year for improved glycemic control, which resulted in bothersome superficial generator migration. Therefore, she underwent the creation of a new subcapsular pocket with the placement of the generator in an absorbable antibiotic pouch. The subcapsular pocket was created just wide enough to accommodate the generator and pouch.

**Figure 1:**
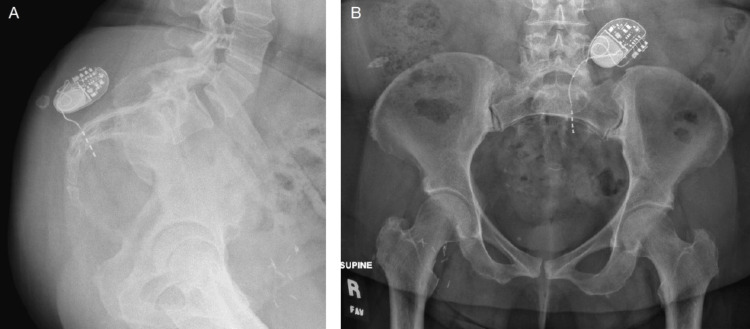
Pre-operative pelvic radiographs A) Lateral view demonstrates posterior lead migration. B) Anterior-posterior (AP) view demonstrates lead twisting near the generator.

Over one year later, she experienced additional intentional 20-pound weight loss as well as two ground-level falls followed by symptom recurrence. Of note, her HbA1C improved to 7.1% at this time with weight loss and adjustments to the medication regimen. A pelvic radiograph demonstrated lead twisting with posterior (retrograde) lead migration consistent with TS (Figure 1). Electrode one was located on the anterior aspect of the sacrum without evidence of lead fracture.

**Figure 2:**
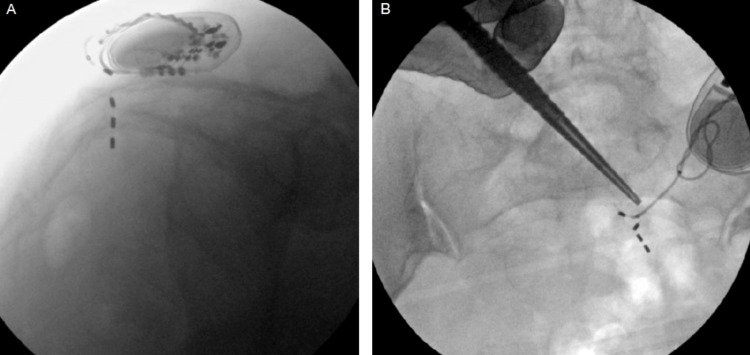
Scout pelvic radiographs prior to surgery A) Lateral view demonstrates worsened lead coiling and further posterior lead migration compared to pre-operative radiographs. B) AP view demonstrates lead fracture between electrodes two and three.

Surgical intervention for revision was delayed by six months due to the patient's preference. The patient denied any further trauma or falls during this time. She also denied any intentional manipulation of the generator. Scout pelvic radiographs were taken at the time of surgery, which demonstrated worsened lead coiling with further posterior lead migration (Figure 2).

**Figure 3:**
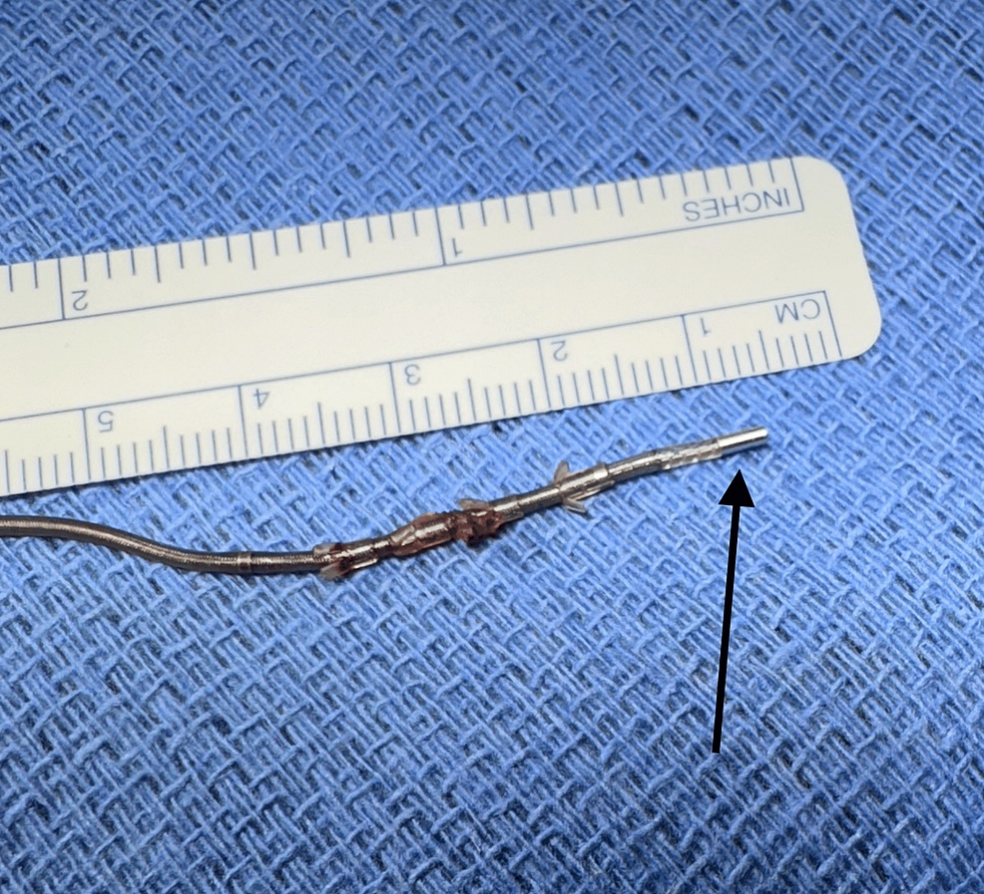
The lead segment removed during the procedure Tined lead segment fractured distal to electrode three (black arrow pointing at electrode 3).

**Figure 4:**
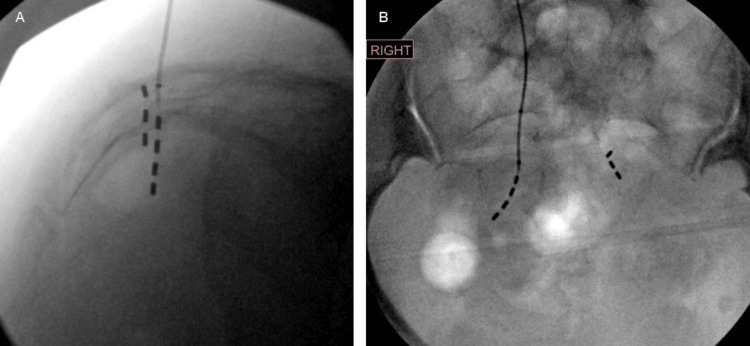
Final pelvic radiographs after lead removal and replacement as well as retained distal lead segment A) Lateral view demonstrates the retained distal lead segment within the sacrum. B) AP view of the new lead on the contralateral side

More notably, the lead was completely fractured between electrodes two and three. Upon accessing the generator, the lead was found to be severely twisted. The proximal portion of the lead was able to be removed under direct vision (Figure 3). However, the distal end of the fractured lead appeared localized within the S3 foramen on X-ray. It was unable to be visualized despite blunt and sharp dissection, so the decision was made to leave the segment of fractured lead in place to avoid any neurovascular injury with further dissection. A new lead was placed on the contralateral S3 foramen with excellent bellows contraction and plantar toe flexion (Figure 4). The previous pocket was revised to a subcapsular location. The generator was found to have 20% battery life pre-operatively, so a new generator was placed inside an antibiotic pouch. Both the generator and pouch were fixed within the revised subcapsular pocket by placing separate absorbable sutures within the two device holes and anchoring them to the underlying tissue. The patient did well post-operatively, with improvement of symptoms at the three-week follow-up.

## Discussion

Twiddler’s syndrome is the voluntary or involuntary manipulation of an implanted device, which may result in device malfunction due to lead migration and potentially lead fracture. Twiddler’s syndrome occurs in 0.07%-1.2% of cardiac pacemakers, though there is currently no data available regarding the prevalence of sacral neuromodulation [1,8,9]. Lead fracture due to TS has been described in pacemakers, vagal nerve stimulators, and baroreflex activator therapy [3,10,11]. To the best of our knowledge, this is the first reported case of TS resulting in a lead fracture in sacral neuromodulation.

The etiology of TS is likely multifactorial with known predictors including female sex, younger age of implantation, use of antipsychotic or antidepressant medication, and higher body mass index [1,12]. Suggested causative factors have been categorized into psychologic characteristics, a technique for implantation, repeated stereotypical movements, and external factors including trauma or infection [12]. Our patient underwent significant intentional weight loss during her time with sacral neuromodulation, which resulted in bothersome superficial generator migration requiring pocket revision. Additionally, the reduction of subcutaneous adipose tissue, as well as a decrease in elastic fibers and newly formed collagen in the skin after weight loss likely contribute to increased generator mobility within the altered capsule [13,14]. Our patient experienced two previous falls, which led us to obtain the initial radiographs that demonstrated lead migration without evidence of lead fracture. Trauma can trigger an inflammatory response and create the formation of a seroma or hematoma, which has been suggested to increase device rotation [5]. In our patient, changes in tissue elasticity and surrounding inflammation associated with weight loss and trauma likely resulted in increased generator mobility. Although our patient denied intentional device manipulation, TS often occurs subconsciously and is the suspected cause of device malfunction in this case given the extensive degree of lead twisting. We suspect that inadvertent device manipulation may worsen as generator mobility increases.

Preventive techniques should be applied to reduce the incidence of TS, particularly among those with known risk factors. Antibiotic pouches and suture fixation of generators have been described as techniques to limit generator movement [8,15]. The use of a nonabsorbable antibiotic pouch in pacemakers and defibrillators significantly reduced “retwiddling” events compared to a suture fixation-only group among patients with known TS [15]. The authors suggest that a pouch helps secure the device by promoting tissue ingrowth and pocket contraction. In our practice, we began to place the generator within an absorbable antibiotic pouch and anchor both together to the underlying tissue with an absorbable suture to limit mobilization during the period of capsule formation. Additional considerations that should be implemented in every patient include limiting the pocket size and placing the generator more superiorly along the iliac crest to avoid involuntary generator manipulation during stereotypic movements [4-7,14]. Psychiatric assessment has been previously recommended for those with psychiatric disorders leading to frequent device manipulation [16]. In cases of TS requiring revision, the tined lead should be replaced as the device torsion clearly causes damage to the lead integrity as evidenced by this case of lead fracture.

## Conclusions

Twiddler’s syndrome, resulting in lead fracture, can also occur in sacral neuromodulation. Preventive techniques may be applied to patients with known risk factors for TS. In cases of TS requiring revision, the lead should be replaced to prevent future lead fracture due to damage from excessive coiling. This case report presents the case of a 57-year-old female who presented with fecal incontinence and refractory overactive bladder and was identified as a case of TS resulting in a lead fracture in sacral neuromodulation. She subsequently underwent partial lead removal and replacement with additional measures to prevent Twiddler’s syndrome and its sequelae.
